# 一种基于疏水基团标记和反相色谱分离的富集策略及其在含赖氨酸多肽分析中的应用

**DOI:** 10.3724/SP.J.1123.2024.02017

**Published:** 2024-07-08

**Authors:** Yu HE, Yichu SHAN, Lihua ZHANG, Zhenbin ZHANG, Yang LI

**Affiliations:** 1.宁波大学新药技术研究院,浙江 宁波 315211; 1. Institute of Drug Discovery Technology, Ningbo University, Ningbo 315211, China; 2.中国科学院大连化学物理研究所,中国科学院分离分析化学重点实验室,辽宁 大连 116023; 2. Key Laboratory of Separation Science for Analytical Chemistry, Dalian Institute of Chemical Physics, Chinese Academy of Sciences, Dalian 116023, China

**Keywords:** 疏水基团标记, 富集, 液相色谱-串联质谱, 含赖氨酸多肽, hydrophobic tagging, enrichment, liquid chromatography-tandem mass spectrometry (LC-MS/MS), lysine-containing peptides

## Abstract

赖氨酸(K)已被广泛用于靶向赖氨酸的交联剂设计、蛋白质复合物的结构解析以及蛋白质-蛋白质相互作用等研究领域。在基于液相色谱-串联质谱(LC-MS/MS)联用技术的“鸟枪法”蛋白质组学研究中,复杂生物样品中的蛋白质被酶切成上万条肽段,为直接分析含有K的肽段带来了巨大挑战。鉴于目前缺乏针对含K多肽的有效富集方法,本工作发展了一种基于疏水标记试剂C10-S-S-NHS和反相色谱分离的方法(简称HYTARP),实现对复杂样品中含K多肽的高效富集和鉴定。合成的C10-S-S-NHS试剂可以高效标记含不同数目K的标准肽段,且对HeLa细胞蛋白酶解肽段的标记效率高达96%。通过考察标记肽段在反相色谱中的保留行为,发现大部分被标记的含K肽段在流动相中乙腈比例升高至约57.6%(v/v)时开始洗脱。进一步优化色谱洗脱梯度,发现阶梯式洗脱能够实现对复杂样品酶解肽段中标记的K肽段的高效分离和富集。富集后样品中含K肽段的占比>90%,较富集前提高了35%。富集的含K肽段对应蛋白质的丰度动态范围跨越了5~6个数量级,实现了对复杂样品中低丰度蛋白质的鉴定。综上,本工作发展的HYTARP策略为降低样品复杂度、提高含K肽段及低丰度蛋白质的鉴定覆盖率提供了一种简单、高效的方法。

## Abstract (en)

Lysine (K) is widely used in the design of lysine-targeted crosslinkers, structural elucidation of protein complexes, and analysis of protein-protein interactions. In “shotgun” proteomics, which is based on liquid chromatography-tandem mass spectrometry (LC-MS/MS), proteins from complex samples are enzymatically digested, generating thousands of peptides and presenting significant challenges for the direct analysis of K-containing peptides. In view of the lack of effective methods for the enrichment of K-containing peptides, this work developed a method which based on a hydrophobic-tag-labeling reagent C10-S-S-NHS and reversed-phase chromatography (termed as HYTARP) to achieve the efficient enrichment and identification of K-containing peptides from complex samples. The C10-S-S-NHS synthesized in this work successfully labeled standard peptides containing various numbers of K and the labeling efficiency achieved up to 96% for HeLa cell protein tryptic digests. By investigating the retention behavior of these labeled peptides in C18 RP column, we found that most K-labeled peptides were eluted once when acetonitrile percentage reached 57.6% (v/v). Further optimization of the elution gradient enabled the efficient separation and enrichment of the K-labeled peptides in HeLa digests via a stepwise elution gradient. The K-labeled peptides accounted for 90% in the enriched peptides, representing an improvement of 35% compared with the number of peptides without the enrichment. The dynamic range of proteins quantified from the enriched K-containing peptides spans 5-6 orders of magnitude, and realized the detection of low-abundance proteins in the complex sample. In summary, the HYTARP strategy offers a straightforward and effective approach for reducing sample complexity and improving the identification coverage of K-containing peptides and low-abundance proteins.

在蛋白质组学研究领域,由Yates提出的“鸟枪法”(shotgun)已被广泛用于蛋白质组的定性定量、蛋白质的翻译后修饰以及蛋白质-蛋白质相互作用等分析中^[1⇓⇓⇓-5]^。该方法首先将蛋白质混合物酶解成多肽,再利用液相色谱-串联质谱(LC-MS/MS)技术对多肽进行分离和鉴定。然而,复杂样品中的蛋白质丰度是高度动态的(高达9个数量级),蛋白质酶解成肽段后进一步增加了样品的复杂程度,高丰度蛋白质/肽段会严重影响对低丰度蛋白质/肽段的鉴定^[6]^。因此,对复杂样品中的蛋白质/肽段直接进行LC-MS/MS分析难以实现对蛋白质组的深度覆盖。

为了解决这个问题,对蛋白质酶解肽段中含有某类特征氨基酸残基的多肽进行富集和分析是降低样品复杂度、提高蛋白质组鉴定覆盖度的有效手段。目前已经发展了针对多种氨基酸残基的多肽富集策略,如半胱氨酸^[7⇓-9]^、赖氨酸(K)^[10,11]^、色氨酸^[12,13]^、甲硫氨酸^[14]^、组氨酸^[15]^以及N端丝氨酸、苏氨酸、脯氨酸、甘氨酸^[16⇓-18]^等。这些方法主要利用可以特征标记目标氨基酸残基的活性基团,并将其共价键合于固相功能材料,从而实现对含特征氨基酸蛋白质/多肽的选择性富集。由于大部分蛋白质(超过96%)含有半胱氨酸残基,Li等^[7]^开发了基于固相烷基化硅胶材料的蛋白质组样品制备方法,该方法对含有半胱氨酸残基蛋白质的富集效率高达93%,具有较高的通量和抗基质干扰能力,提高了临床蛋白质样品的回收率。Qi等^[10]^发展了一种固定化蛋白质的酶解方法,利用可以选择性标记氨基的三氟代功能化微球对蛋白质进行快速固定和高效酶解,蛋白质回收率>90%,显著提高了复杂样品中肽段的鉴定数目和蛋白质序列覆盖率。类似地,Shah等^[11]^将可与氨基反应的醛基基团键合于固相材料,并用于福尔马林固定和石蜡包埋组织样品的蛋白质固定、干扰物去除和蛋白质酶解,蛋白质回收率高达95%。为了降低样品复杂度,Li等^[17]^开发了一种选择性富集N末端脯氨酸肽段的方法,首先采用邻苯二甲醛封闭酶解肽段的伯胺基团,再采用戊二醛在还原环境下标记肽段N末端的脯氨酸残基——仲胺,利用固相酰肼微球选择性富集标记后的N末端脯氨酸肽段,该方法对N末端脯氨酸肽段的富集效率高达93.7%,提高了复杂样品中低丰度蛋白质的鉴定。以上方法均采用了功能化材料富集具有特定氨基酸残基的蛋白质/肽段样品,然而由于蛋白质复杂的空间结构引入的空间位阻效应和固-液反应效率低等原因,样品中的蛋白质难以被完全捕获。此外,功能化材料表面存在大量的非特异性吸附位点,导致样品损失和引入杂质。

K由于在蛋白质中分布较广(约6%),侧链氨基反应特异性高且广泛分布在溶剂可接触的蛋白质表面,已被广泛用于K靶向的交联剂设计、蛋白质复合物的结构解析以及蛋白质-蛋白质相互作用等研究领域^[19⇓-21]^。能够与K侧链的伯胺发生反应的试剂众多,包括卤化物^[10]^、醛类^[11]^、亚胺酸酯类^[22]^、胍基类^[23]^和琥珀酰化类^[24]^等。其中,琥珀酰化类试剂由于反应特异性高、反应条件温和、生成物结构稳定而被广泛用于化学交联蛋白质复合物的结构及其相互作用研究^[25,26]^。最常用的化学交联试剂,如双琥珀酰亚胺辛二酸酯(DSS)、二(磺基琥珀酰亚胺)辛二酸酯(BS^3^)和双琥珀酰亚胺亚砜(DSSO)^[27]^,均是由*N*-羟基丁二酰亚胺(NHS)官能团组成的氨基靶向试剂。然而,针对含K蛋白质/肽段的选择性富集方法却鲜有报道。一方面由于蛋白质的K残基被大部分试剂标记后,由于空间位阻效应,胰蛋白酶无法酶切标记后的K位点,导致酶解肽段过长,不利于MS鉴定^[28]^;另一方面可能由于大部分氨基反应试剂可以同时标记蛋白质/肽段N末端的*α*-氨基与K侧链的*ε*-氨基,导致富集过程中含K多肽与其他不含K多肽难以分离。

针对目前功能化富集材料存在的不足以及缺乏可以有效富集含K多肽的方法,本工作发展了一种基于疏水基团标记和反相色谱分离的方法(hydrophobic tagging and reversed-phase chromatography separation,简称HYTARP),以实现对复杂样品中含K多肽的高效富集和鉴定。首先利用合成的标记试剂2,5-二氧代吡咯烷-1-基-3-(癸基二硫代)丙酸酯(C10-S-S-NHS)对胰蛋白酶酶解肽段中的氨基(包括肽段N末端和K的侧链氨基)进行C10疏水基团标记。由于含K肽段比不含K肽段多标记至少一个C10烷基链,其在反相色谱中的保留将明显增强。通过优化反相色谱的洗脱梯度,实现了对含K肽段的选择性富集。再进一步断裂标记试剂中的二硫键,释放被标记肽段的C10烷基链,保证含K肽段在LC-MS/MS中的高效分离和鉴定。该方法在肽段水平标记含K多肽,避免了胰蛋白酶的漏切问题;并且利用具有高分辨率的反相色谱可以有效提高对含K多肽的富集选择性,同时避免了大量功能化材料的使用,提高了样品回收率。

## 1 实验部分

### 1.1 仪器、材料与试剂

用于反相色谱分离疏水标记多肽的LC-20AD泵和SPD-20A检测器构建的液相色谱仪购自日本岛津公司。Ascend 500 MHz核磁共振仪和Ultraflex Ⅲ型基质辅助激光解吸电离飞行时间(MALDI-TOF)质谱仪购自德国Bruker公司。EASY-nLC 1000色谱仪与Q-Exactive质谱仪联用系统、UltiMate 3000 RSLC色谱仪与Orbitrap Fusion Lumos Tribrid质谱仪联用系统购自美国Thermo Fisher公司;04714-50细胞超声破碎仪购自美国Cole-Parmer公司。用于测定蛋白质浓度的全波长酶标仪购自美国BioTek公司。用于nanoRPLC-ESI-MS/MS分析的熔融石英毛细管(150 μm i. d.×375 μm o. d.)购自美国Polymicro Technologies公司。Reprosil-Pur C18-AQ硅胶填料(1.9 μm, 12 nm)购自德国Dr. Maisch公司。Venusil XBP C18硅胶填料(5 μm, 12 nm)购自天津博纳艾杰尔公司。

尿素(urea)、蛋白酶抑制剂(Cocktail)、三(2-羧乙基)膦盐酸盐(TCEP)、碘乙酰胺(IAA)、4-(2-羟乙基)哌嗪-1-乙磺酸(HEPES)、甲酸(FA)、三氟乙酸(TFA)购自美国Sigma-Aldrich公司。磷酸盐缓冲液(1×PBS)、细胞培养用的MEM培养基、胎牛血清(FBS)和链霉素(10 mg/mL)/青霉素(10 kU/mL)购自美国Gibco公司。胰蛋白酶(trypsin)购自美国Promega公司。2,2'-二吡啶二硫醚和1-癸硫醇购自日本Tokyo Chemical Industry公司。3-巯基丙酸购自上海阿拉丁生化科技股份有限公司。NHS和*N*-(3-二甲氨基丙基)-*N*'-乙基碳二亚胺盐酸盐(EDC·HCl)购自北京百灵威科技有限公司。碳酸氢铵(NH_4_HCO_3_)、二氯甲烷(DCM)购自天津科密欧化学试剂有限公司。乙腈(ACN, HPLC级别)购自德国Merck公司。BCA蛋白质浓度测定试剂盒购自上海碧云天生物技术有限公司。所有实验用水均通过Milli-Q系统(美国Millipore公司)纯化。其他化学品和溶剂均为分析级。所有标准多肽购自上海强耀生物科技有限公司。

### 1.2 标记试剂C10-S-S-NHS的合成

标记试剂C10-S-S-NHS的合成步骤参考文献[29]。主要合成过程如下:(1)氮气保护下,将2,2'-二吡啶二硫醚(3.3 g, 15 mmol)溶于25 mL无水乙醇中,依次加入1.25 mL乙酸和3-巯基丙酸(1.06 g, 10 mmol),室温搅拌过夜。减压旋蒸去除有机溶剂后,用碱性氧化铝层析柱纯化产物,先用DCM-EtOH(体积比3∶2)淋洗,再用DCM-EtOH-醋酸(体积比30∶20∶2)洗脱并冻干,得目标产物3-(2-吡啶二硫代)丙酸(2.1 g,收率98%); (2)氮气保护下,将3-(2-吡啶二硫代)丙酸(2 g, 9.3 mmol)和1-癸硫醇(1.46 g, 8.4 mmol)加入23 mL甲醇中,室温搅拌1 h。减压旋蒸后,用硅胶层析柱纯化产物,先用DCM淋洗,再用DCM-乙酸乙酯(体积比15∶1)洗脱并冻干,得目标产物1-癸烷基二硫代丙酸(淡黄色油状物,1.15 g,产率49%); (3)氮气保护下,将1-癸烷基二硫代丙酸(1.15 g, 4.1 mmol)、 NHS (0.95 g, 8.3 mmol)和EDC·HCl(1.58 g, 8.3 mmol)依次加入18.4 mL DCM中,室温搅拌过夜。饱和食盐水洗涤有机层后,用硫酸钠干燥。减压旋蒸后,用硅胶层析柱纯化产物,用DCM-石油醚(体积比3∶2)洗脱并冻干,即得目标产物C10-S-S-NHS(淡黄色固体,0.72 g,收率46%)。

### 1.3 细胞培养

用含有10% FBS和1%青霉素/链霉素的MEM培养基复苏HeLa细胞。将复苏后的细胞置于37 ℃恒温培养箱中,5% CO_2_气体条件下培养,每隔3天传代一次。细胞用预冷的1×PBS清洗3次后,加入含0.05%胰蛋白酶的EDTA溶液于37 ℃消化5 min,使贴壁细胞从培养皿上脱离,再加入含有血清的培养基终止胰酶消化。收集细胞悬液,于4 ℃以500 g离心3 min收集细胞。最后用预冷的1×PBS清洗细胞3次。

### 1.4 蛋白质提取和酶解

将HeLa细胞悬浮于由8 mol/L尿素和1%(v/v)蛋白酶抑制剂组成的细胞裂解液中(50 mmol/L NH_4_HCO_3_, pH 8.0),并在冰浴下超声破碎60 s(80%能量,超声频率为5 s开,5 s关)。匀浆液在4 ℃下以16000 g离心30 min,收集上清。采用BCA法测定蛋白质浓度。将提取的HeLa细胞蛋白质进行还原(10 mmol/L TCEP, 56 ℃, 1 h)和烷基化(30 mmol/L IAA,室温遮光,30 min)。用50 mmol/L NH_4_HCO_3_溶液(pH 8.0)将样品稀释8倍后,以1∶40(m/m)的酶-蛋白质比例加入胰蛋白酶,37 ℃反应12 h。向溶液中加入终体积分数为1%的TFA,终止酶解反应。将酶解产物上样到自制的C18捕集柱(10 mm×4.6 mm, Venusil XBP C18 硅胶填料)上,去除溶液中过量的盐。流动相A: 2%(v/v)乙腈水溶液(含0.1%(v/v)TFA),流动相B: 98%(v/v)乙腈水溶液(含0.1%(v/v)TFA),流速1 mL/min。梯度设置为阶梯式洗脱程序:0~5 min, 2%B; 5.01~15 min, 80%B;收集在80%B相中不保留的肽段洗脱液,即纯化后的HeLa酶解肽段,冷冻干燥。

### 1.5 疏水基团标记

分别将10 μg标准肽段LVVSTQTALA、MIFVGIK和KSLSLSPGK重溶于10 μL 50 mmol/L HEPES缓冲液(pH 8.3)中,加入40 μL含5 g/L C10-S-S-NHS的DMF溶液,40 ℃反应2 h。将50 μg HeLa酶解肽段重溶于20 μL 50 mmol/L HEPES缓冲液(pH 8.3)中,加入80 μL含12.5 g/L C10-S-S-NHS的DMF溶液,肽段的终质量浓度为0.5 g/L, 40 ℃反应4 h。加入终浓度50 mmol/L NH_4_HCO_3_终止标记反应。

### 1.6 反相色谱分离

采用自制的XBP C18分离柱(250 mm×4.6 mm, 5 μm)对疏水基团标记的HeLa酶解肽段进行分级。流速1 mL/min,紫外检测波长为214 nm。

采用线性洗脱程序时流动相A为2%(v/v)乙腈水溶液(含0.1%(v/v)TFA),流动相B为80%(v/v)乙腈水溶液(含0.1%(v/v)TFA),梯度设置如下:0~15 min, 60%B; 15~25 min, 60%B~70%B; 25~55 min, 70%B~80%B; 55~70 min, 80%B~95%B; 70~80 min, 95%B。

采用阶梯式洗脱程序时流动相A为2%(v/v)乙腈水溶液(含0.1%(v/v)TFA),流动相B为98%(v/v)乙腈水溶液(含0.1%(v/v)TFA),梯度设置如下:0~10 min, 32%B; 10.1~20 min, 60%B; 20.1~30 min, 95%B。依据保留时间,收集肽段流出液,并冷冻干燥。将各个肽段级分重溶于含5 mmol/L TCEP的50 mmol/L NH_4_HCO_3_溶液中,56 ℃反应1 h。

### 1.7 MALDI-TOF MS分析

将1 μL疏水标记前后的标准肽段与1 μL的*α*-氰基-4-羟基肉桂酸基质(CHCA, 7 g/L,溶于含0.1%TFA的60% ACN溶液中)依次点于MALDI靶板上。采用固体激光SmartBeam技术(355 nm)及正离子反射模式进行MS分析。采用FlexControl(3.0)采集MS数据,采用FlexAnalysis(3.0)提取所有信噪比大于3的信号峰进行分析。

### 1.8 nanoRPLC-ESI-MS/MS分析

采用EASY-nLC 1000色谱仪与Q-Exactive质谱仪联用系统对采用反相色谱线性梯度分离的肽段级分进行分析。将肽段级分上样于自制的C18捕集柱(30 mm×150 μm, 5 μm),再通过自制C18分离柱(150 mm×150 μm, 1.9 μm)进行分离,流速为600 nL/min。使用流动相A(2%(v/v)乙腈水溶液(含0.1%(v/v) FA))和B(98%(v/v)乙腈水溶液(含0.1%(v/v)FA))建立75 min线性梯度(0~45 min, 6%B~23%B; 45~65 min, 23%B~40%B; 65~70 min, 40%B~80%B; 70~75 min, 80%B)。喷雾电压为2.4 kV,离子传输管温度为250 ℃。在正离子模式下,采用数据依赖模式(DDA), MS分辨率设为70000,扫描范围为*m/z* 300~1800,自动增益控制(AGC)设为3.0×10^6^,离子最大注入时间(M.I.T.)为60 ms。选择电荷价态从+2到+6的母离子肽段,并采用高能碰撞碎裂模式(HCD)对20个响应最强的母离子进行碎裂,碰撞能量设为28。MS^2^的分辨率设为17500, AGC设为5.0×10^4^, M.I.T.设为60 ms,动态排除窗口时间为12 s。

采用UltiMate 3000 RSLC色谱仪与Orbitrap Fusion Lumos Tribrid质谱仪构建的纳升级系统对采用反相色谱阶梯式梯度分离的肽段级分进行分析。将肽段上样于C18捕集柱(20 mm×75 μm, 5 μm ),再通过C18分离柱(250 mm×75 μm,1.9 μm)进行分离,流速为350 nL/min。使用流动相A(2%(v/v)乙腈水溶液(含0.1%(v/v)FA))和B(80%(v/v)乙腈水溶液(含0.1%(v/v)FA))建立60 min线性梯度(0~6 min, 2%B~5%B; 6~43 min, 5%B~18%B; 43~48 min, 18%B~22%B; 48~51 min, 22%B~35%B; 51~54 min, 35%B~95%B; 54~60 min, 95%B)。喷雾电压为2.1 kV,离子传输管温度为320 ℃。在正离子模式下,采用DDA模式每3 s完成一次全扫描循环。选择轨道阱(OT)采集MS数据,分辨率设为60000。扫描范围为*m/z* 350~1800, AGC设为4.0×10^5^, M.I.T.为50 ms。选择电荷价态从+1到+7的母离子肽段,并采用HCD模式对其进行碎裂,碰撞能量设为30。OT采集MS^2^数据,分辨率设为15000, AGC设为5.0×10^4^, M.I.T.设为30 ms,动态排除窗口时间为20 s。

### 1.9 数据分析

将质谱数据文件(*.raw)采用嵌入了Mascot(版本2.3.2)搜索引擎的Proteome Discoverer(PD)(版本2.1.1.21)软件进行二级质谱数据库搜索,数据库为从Uniprot网站下载的人源蛋白质数据库(共20360个蛋白质条目)。检索参数设置如下:胰蛋白酶完全酶切,最多允许2个漏切位点;固定修饰为半胱氨酸的还原烷基化,可变修饰有K和肽段N末端的硫酰化,蛋氨酸的氧化和蛋白质N末端的乙酰化;一级母离子质量允许偏差为10×10^-6^(10 ppm),二级碎片离子的质量偏差为0.02 Da。检索结果控制蛋白质和肽段的假阳性率不大于1%;蛋白质鉴定至少包含一个唯一性肽段。采用MaxLFQ算法结合MaxQuant(version 1.6.5.0)搜索引擎用于蛋白质的无标记定量分析,搜库条件与PD保持一致。

## 2 结果与讨论

### 2.1 基于HYTARP策略的富集流程

基于HYTARP策略富集含K多肽的流程如图1所示,首先采用试剂C10-S-S-NHS对胰蛋白酶酶解肽段中K侧链的自由氨基进行疏水基团C10烷基链的标记,同时肽段N末端的自由氨基也会被标记。采用反相色谱对标记后的肽段进行分离,由于C10疏水链的引入,酶解肽段在C18柱上的保留较标记前将整体增强。通过优化反相色谱的分离梯度,实现对含K肽段的选择性富集。再通过二硫键还原反应,释放K侧链和肽段N末端的疏水基团C10烷基链,保证肽段的离子化效率,从而提高nanoLC-MS/MS对含K肽段的分析和鉴定能力。

**图1:**
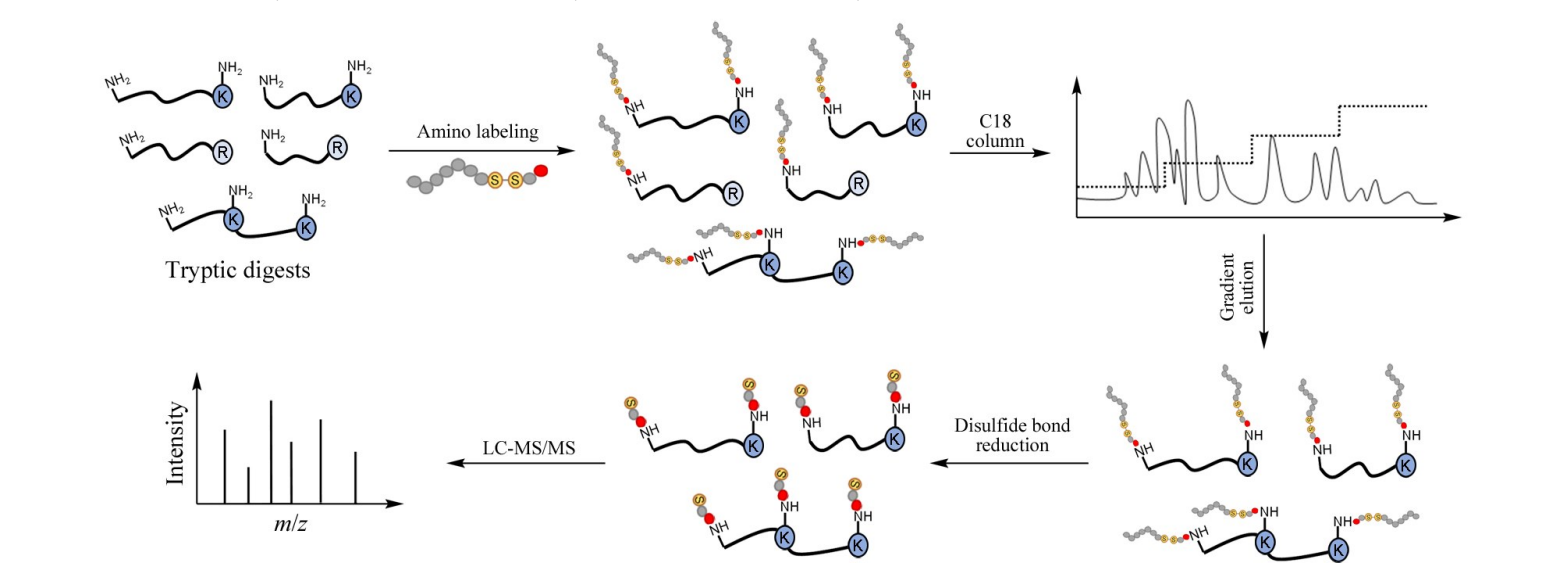
基于HYTARP策略的含K多肽的富集流程示意图

C10-S-S-NHS的合成路线如图2所示(C10-S-S-NHS及中间产物的核磁共振氢谱表征见附图1,www.chrom-China.com),其主要包含3个功能基团:可以高效且特异标记酶解肽段中游离氨基的NHS、可以提高肽段疏水性的长碳链(C10)以及通过还原反应释放C10疏水链的二硫键(S-S)。

**图2:**
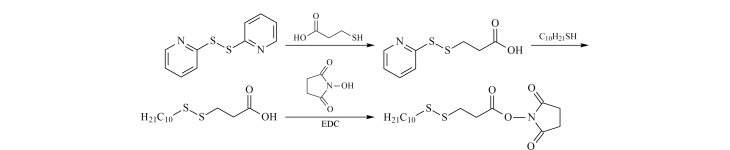
标记试剂C10-S-S-NHS的合成

### 2.2 标记效率考察

为了考察C10-S-S-NHS的标记效率,选取3条含有不同数目K的标准肽段,包括不含K的肽段LA-10(序列LVVSTQTALA),含有一个K的肽段MK-7(序列MIFVGIK),以及含有两个K的肽段KK-9(序列KSLSLSPGK)。采用MALDI-TOF MS对标记前后肽段的相对分子质量进行分析,结果如图3所示,标记后原始的LV-10肽段质谱峰(*m/z* 1024.8,图3a)消失,同时产生新的肽段质谱峰(*m/z* 1284.5,图3b),增加了一个C10链的相对分子质量(Δ*m*=260 Da),表明LV-10肽段的N末端氨基被C10-S-S-NHS完全标记。同样地,标记后的MK-7和KK-9的原始肽段(*m/z* 807.7,图3c; *m/z* 916.7,图3e)都几乎消失,且新生成的肽段分别被标记上2个和3个C10链(*m/z* 1350.2,图3d; *m/z* 1720.1,图3f,+Na峰),表明C10-S-S-NHS试剂可以高效标记肽段N末端和K侧链的自由氨基。

**图3:**
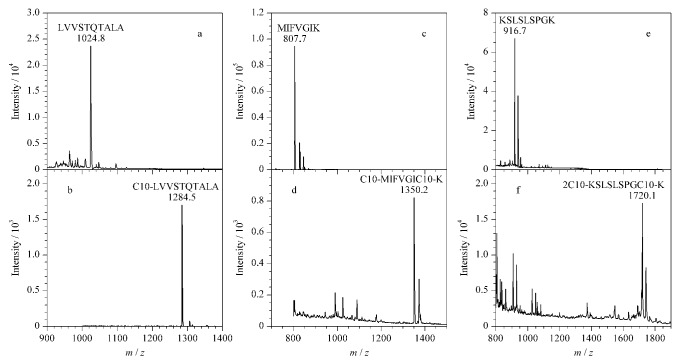
标准肽段被C10-S-S-NHS标记前后的MALDI/TOF MS图

进一步考察了C10-S-S-NHS对复杂样品HeLa酶解肽段的标记效率。将二硫键断裂释放C10链后,标记肽段上的氨基生成一个硫酰基(-COCH_2_CH_2_SH)修饰。通过统计标记后肽段样品中氨基被硫酰基修饰的比例,得到C10-S-S-NHS对HeLa酶解肽段的标记效率为96.2%,其中被标记1个、2个、3个C10链的肽段比例分别为46.6%、49.1%、0.6%(图4)。分别选取被标记1个、2个、3个C10链肽段的MS/MS图(附图2),发现肽段的N末端和K侧链的氨基均被标记上硫酰基,且匹配到了大部分b、y离子,表明标记后的含K多肽可以被MS高效鉴定。值得一提的是,在3.8%未被标记的酶解肽段中,有85.7%的氨基位于蛋白质的N末端且发生了乙酰化修饰,进一步说明了C10-S-S-NHS对复杂样品中自由氨基的高效标记,为含K多肽与不含K多肽在后续反相色谱中的有效分离提供保障。

**图4:**
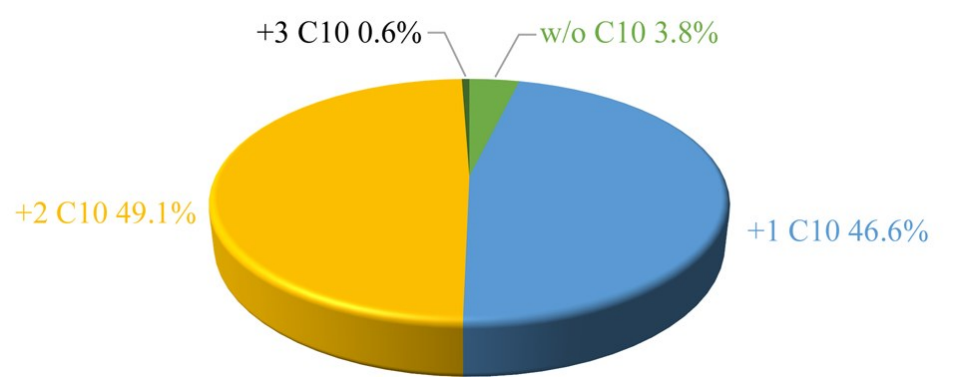
HeLa酶解肽段标记0、1、2和3个C10烷基链的百分比分布

### 2.3 分离梯度考察

将HeLa酶解肽段采用C10-S-S-NHS进行疏水基团标记后进行反相色谱分离。首先采用线性梯度洗脱,收集不同保留时间的肽段流出液,并将二硫键还原释放C10链后,进行nanoLC-MS/MS分析。色谱分离图如图5a所示,由于标记C10链后酶解肽段的疏水性整体增强,选择流动相中ACN含量为48% (v/v)作为初始洗脱浓度(即60%B,流动相B为80%(v/v)乙腈水溶液),此时样品中不保留的肽段主要为未被C10标记和N端被标记一个C10(即不含K)的肽段(占比99.1%, 0~17 min,图5b);当升高ACN浓度至约57.6%(72%B, 35 min),不保留肽段开始以被标记2个及以上C10的含K肽段为主(>50%),并且含K肽段的比例随着洗脱浓度升高呈递增趋势。当ACN浓度增加至62.4%(78%B, 48 min)时含K肽段的比例>90%。样品中的标记试剂C10-S-S-NHS由于过量且疏水性较强,在约46 min时产生较强的紫外吸收峰;部分C10-S-S-NHS水解后,疏水的NHS基团变成亲水的羧基(COOH),由于疏水性降低,在C18色谱柱中的保留前移了8 min(约38 min)。综上,以48% ACN(v/v)为初始洗脱浓度,此时未被C10标记的肽段可以被完全洗脱;逐渐升高洗脱浓度至约57.6% ACN,大部分N端被标记一个C10的不含K肽段被洗脱,此后洗脱的肽段以被标记2个C10及以上的含K肽段为主,以上结果表明含K肽段在反相色谱中可以与不含K肽段实现有效分离。

**图5:**
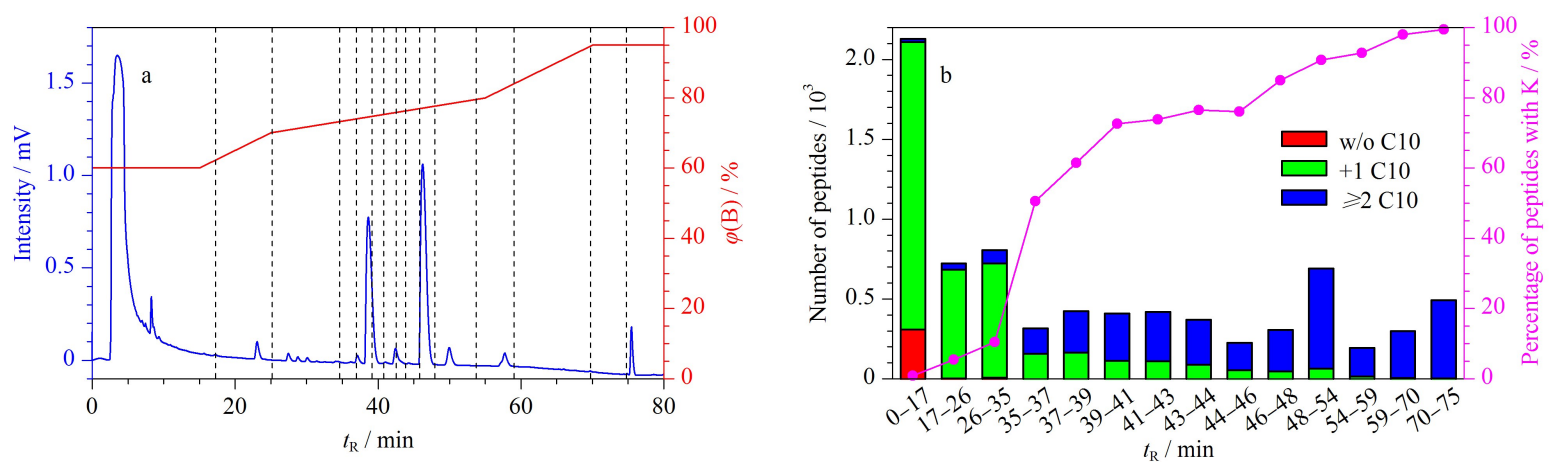
(a) HeLa酶解肽段被C10-S-S-NHS标记后的反相分离色谱图, (b)各级分中被标记0个、1个和≥2个 C10链的肽段数(堆积柱形图)以及含K肽段所占百分比(折线图)

根据上述考察的分离梯度,进一步考察采用阶梯式梯度的洗脱方式对HeLa酶解肽段中含K多肽的富集效果。流动相A为2%(v/v)乙腈水溶液(含0.1%(v/v)TFA),流动相B为98%(v/v)乙腈水溶液(含0.1%(v/v)TFA)。4个梯度设置如下, #1: 0~10 min, 32%B; 10.1~20 min, 56%B; 20.1~30 min, 95%B; #2: 0~10 min, 32%B; 10.1~20 min, 60%B; 20.1~30 min, 95%B; #3: 0~10 min, 32%B; 10.1~20 min, 64%B; 20.1~30 min, 95%B; #4: 0~10 min, 32%B; 10.1~20 min, 68%B; 20.1~30 min, 95%B。

收集4个洗脱梯度下的不保留肽段馏分,还原二硫键释放C10链后,进行nanoLC-MS/MS分析。如附图3a所示,在32%B洗脱条件下,鉴定到的肽段以未被C10标记的肽段为主,其中N端被乙酰化等修饰的肽段占比88.9%,含K肽段仅占0.4%。当B相浓度分别提高至56%~68%(#1~#4),鉴定到的肽段均以N端被标记1个C10的肽段为主(>97.5%)。当继续提高B相浓度至95%(附图3b),鉴定到的肽段以被标记2个及以上C10的含K肽段为主,且含K肽段的比例在#3条件下最高(占比85.9%),其次为#2、#1和#4(含K肽段占比分别为84.7%、74.3%和72.5%),以上结果说明采用阶梯式洗脱梯度可以实现对复杂酶解肽段样品中含K多肽的有效富集。由于鉴定到的含K肽段在#2条件下最多,后续将采用#2的洗脱梯度作为含K多肽的富集条件。

### 2.4 HeLa酶解肽段中含K多肽的富集及分析

综上,为了实现对HeLa酶解肽段中含K多肽的高效富集,将HeLa酶解肽段进行C10-S-S-NHS疏水基团标记后,采用#2阶梯式梯度进行反相色谱分离,收集不同洗脱浓度下的不保留肽段级分,将标记肽段上的二硫键还原释放C10链后,进行nanoLC-MS/MS分析。由于氨基被硫酰基修饰,肽段的带电数目可能降低。为了提高对含K肽段的鉴定覆盖度,在质谱采集时将携带+1价及以上电荷的母离子进行二级碎裂。鉴定结果如图6a所示,在32% ACN洗脱条件下,不保留肽段以未被C10标记的肽段为主(占比95.7%),其中含K肽段仅占0.04%。当洗脱梯度升高至60% ACN,高达99.0%肽段为N端被标记一个C10的不含K肽段,含K肽段比例仅为0.6%。继续升高梯度至95% ACN,不保留肽段以被标记2个及以上C10的含K肽段为主,占比为90.2%,较富集前提高了35%。在平行两次的95% ACN中共鉴定到4568条含K肽段,序列重合度为62.9%(附图4)。对不同洗脱梯度下鉴定到的含K肽段的序列重合度进行比较(图6b),发现95% ACN下单独鉴定到的含K肽段占总含K肽段的99.0%(4561/4606),表明高分辨率的反相色谱对复杂样品酶解肽段中的含K肽段实现了高效分离和富集。进一步对富集的含K肽段的序列进行特征分析,如图6c所示,K位点附近的氨基酸分布以酸性氨基酸谷氨酸和侧链为非极性的氨基酸丙氨酸、亮氨酸为主,反映了含K肽段具有一定的序列一致性。对富集的含K肽段定量到的蛋白质丰度分布进行分析,如图6d所示,含K肽段定量到的蛋白质丰度动态范围(红色圆圈)跨越了5~6个数量级,几乎覆盖了全部肽段定量到的所有蛋白质(蓝色圆圈),且能够鉴定到更多低丰度的蛋白质。

**图6:**
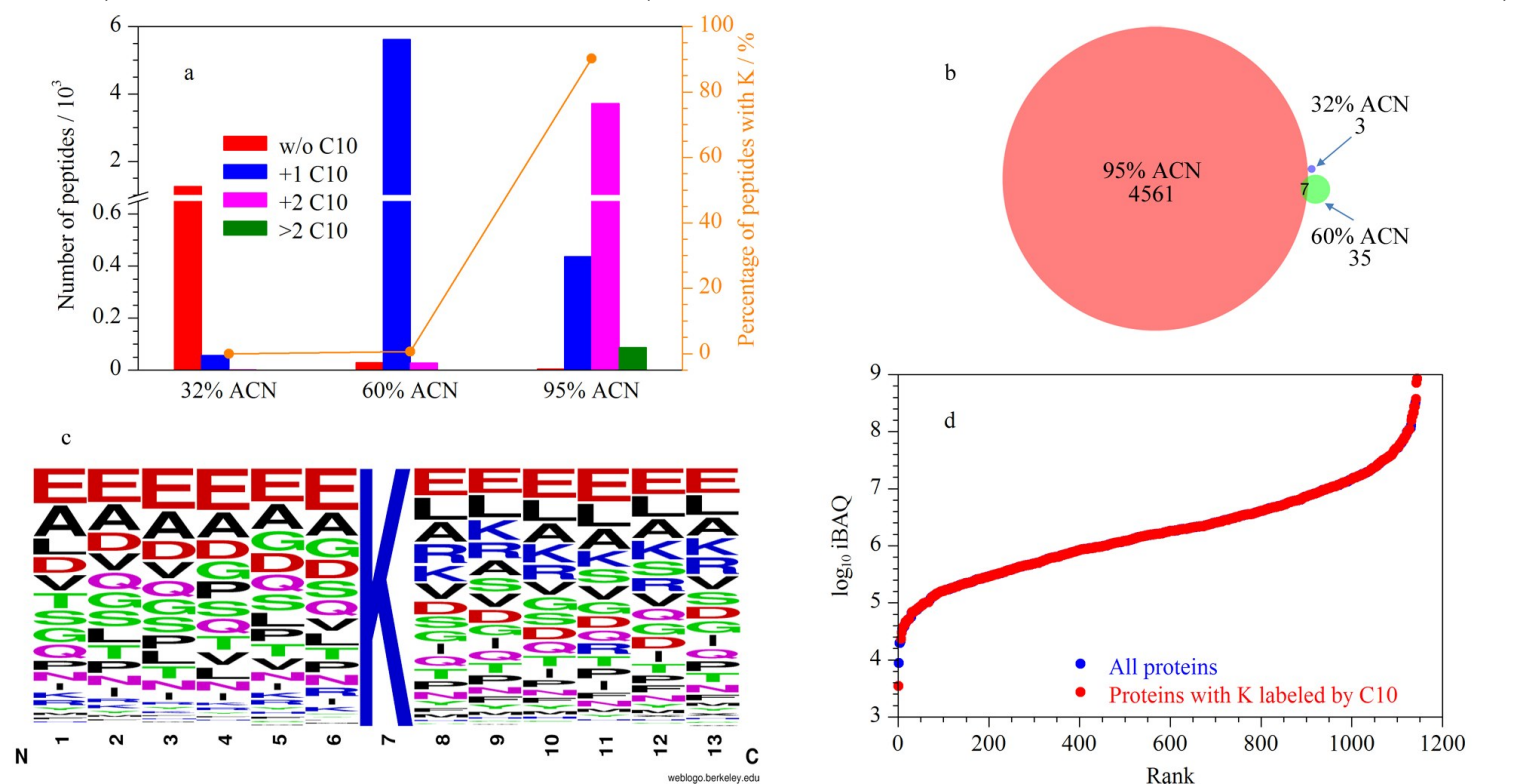
(a)各级分中被标记0个、1个、2个和2个以上C10链的肽段数(堆积柱形图)以及含K肽段所占百分比(折线图), (b)各级分间含K肽段的鉴定结果Venn图,(c)富集的含K肽段的序列特征,(d)定量到的全部(蓝色圆圈)以及含有被C10标记的K的(红色圆圈)Hela细胞蛋白质的丰度范围

## 3 结论

本工作发展了一种基于疏水基团标记和反相色谱分离的方法,对复杂样品中含K多肽进行高效富集和鉴定。合成的试剂C10-S-S-NHS对HeLa细胞酶解肽段的标记效率高达96%。标记后的含K肽段与不含K肽段在反相色谱实现了高效分离。富集后样品中含K肽段的比例高达90%,较富集前提高了35%,对应蛋白质的丰度动态范围几乎覆盖了全部肽段定量到的蛋白质,且能够鉴定到更多低丰度的蛋白质。本文发展的HYTARP策略为降低样品复杂度,提高对含K肽段和低丰度蛋白质的鉴定覆盖率提供了一种简单、高效的方法。
